# Insulin pump treatment vs. multiple daily insulin injections in patients with poorly controlled Type 2 diabetes mellitus: a comparison of cardiovascular effects

**DOI:** 10.1007/s12020-023-03651-w

**Published:** 2024-01-10

**Authors:** Saverio Tremamunno, Linda Tartaglione, Alessandro Telesca, Alessandro Rizzi, Tamara Felici, Francesco Mazzotta, Antonio De Vita, Emanuele Rizzo, Nello Cambise, Antonietta Belmusto, Dario Pitocco, Gaetano Antonio Lanza

**Affiliations:** 1 Department. of Cardiovascular Sciences, Fondazione Policlinico Universitario A. Gemelli IRCCS, Università Cattolica del Sacro Cuore, Rome, Italy; 2 Diabetes Care Unit, Internal Medicine, Fondazione Policlinico Universitario A. Gemelli IRCCS, Università Cattolica del Sacro Cuore, Rome, Italy

**Keywords:** Type 2 diabetes mellitus, Continuous subcutaneous insulin injection, Continuous glucose monitoring, Flow-mediated dilatation, Heart rate variability

## Abstract

**Aims:**

Both hyperglycaemia and large glycaemic variability are associated with worse outcomes in patients with Type 2 diabetes mellitus (T2DM), possibly causing sympatho-vagal imbalance and endothelial dysfunction. Continuous subcutaneous insulin injection (CSII) improves glycemic control compared to multiple daily insulin injections (MDI). We aimed to assess whether CSII may improve cardiac autonomic and vascular dilation function compared to MDI.

**Methods:**

We enrolled T2DM patients without cardiovascular disease with poor glycaemic control, despite optimized MDI therapy. Patients were randomized to continue MDI (with multiple daily peripheral glucose measurements) or CSII; insulin dose was adjusted to achieve optimal target ranges of blood glucose levels. Patients were studied at baseline and after 6 months by: 1) flow-mediated dilation (FMD) and nitrate-mediated dilation (NMD) of the brachial artery; 2) heart rate variability (HRV) by 24-hour ECG Holter monitoring (HM). 7-day continuous glucose monitoring (CGM) was performed in 9 and 8 patients of Group 1 and 2, respectively.

**Results:**

Overall, 21 patients were enrolled, 12 randomized to CSII (Group 1) and 9 to MDI (Group 2). The daily dose of insulin and Hb1AC did not differ significantly between the 2 groups, both at baseline and at follow-up. Glucose variability showed some significant improvement at follow-up in the whole population, but no differences were observed between the 2 groups. Both FMD and NMD, as well as HRV parameters, showed no significant differences between the 2 groups at 6-month follow-up.

**Conclusions:**

In this randomized small study we show that, in T2DM patients, CSII achieves a similar medium-term glycemic control compared to MDI, without any adverse effect on the cardiovascular system.

## Introduction

Hyperglycaemia is a predictor of long-term prognosis in patients with diabetes mellitus (DM), favouring the development and progression of macrovascular and microvascular complications [1–4]. On the other hand, hypoglycaemic episodes, and extensive variability in glucose blood levels, have also been associated with a negative clinical outcome in patients with DM [5–7], possibly by causing sympatho-vagal imbalance, which negatively affects the cardiovascular system [8–11]. In a previous study, we showed that, in Type 2 DM (T2DM) patients with coronary artery disease (CAD), hypoglycaemic episodes were associated with a marked reduction of heart rate variability (HRV) [12].

Prior studies have also shown that T2DM is associated with a significant early impairment of endothelial dilation function, which is believed to constitute a significant risk factor for the development of both macrovascular atherosclerotic complications as well as microvascular complications [13–15]. Thus, the reduction of both hyperglycaemic and hypoglycaemic episodes, as well as glucose variability (GV), might have favourable outcomes on the cardiovascular system and clinical benefit in T2DM patients. Recent data suggested that insulin pump treatment (continuous subcutaneous insulin infusion/CSII) improves glycemic control in patients with poorly controlled T2DM compared to MDI. [16, 17].

Thus, in this study we aimed to evaluate whether use of CSII may improve cardiac autonomic function and/or systemic vascular dilation function compared to multiple daily injections (MDI) and whether its possible effects are associated with a better control of glycaemic levels and GV [18, 19].

## Methods

### Inclusion and exclusion criteria

From June 2018 to May 2020, we enrolled consecutive T2DM patients aged between 30 and 75 years, on MDI with or without additional anti-diabetic drugs, referred to our Diabetic Centre with poorly controlled glycaemia, defined as HbA1c levels between 8.0% and 12.0% (64 to 108 mmol/mol), despite a daily dose of insulin of 0.7–1.8 units/kg or maximum dose of 220 units and >2 blood glucose self-assessments per day.

Patients with any of the following clinical conditions were excluded: 1) overt cardiovascular disease, i.e., any documented evidence of known cardiac, cerebral or peripheral artery disease, as well as any symptoms or abnormal findings at physical examination or non-invasive diagnostic tests (including electrocardiogram and cardiac and arterial ultrasound studies) indicating cardiovascular disease; 2) presence of serious medical conditions, including liver cirrhosis, malignancies, acute or chronic inflammatory disease; 3) pregnancy; 4) psychological conditions that might have hampered patient’s compliance; 5) two or more symptomatic hypoglycaemia episodes in the past 6 months; 6) presence of significant diabetic microvascular complications (i.e., diabetic retinopathy or neuropathy, or chronic kidney disease, as indicated by an estimated glomerular filtrate rate lower than 15 ml/min/1.73 m^2^); 7) any other condition that, in the judgment of the investigators, precluded the participation of the patient in the study. Furthermore, in all patients included in the study, autoimmune diabetes was excluded by measuring anti-glutamic acid decarboxylase and anti-tyrosine phosphatase (IA-2) antibodies.

**Fig. 1:**
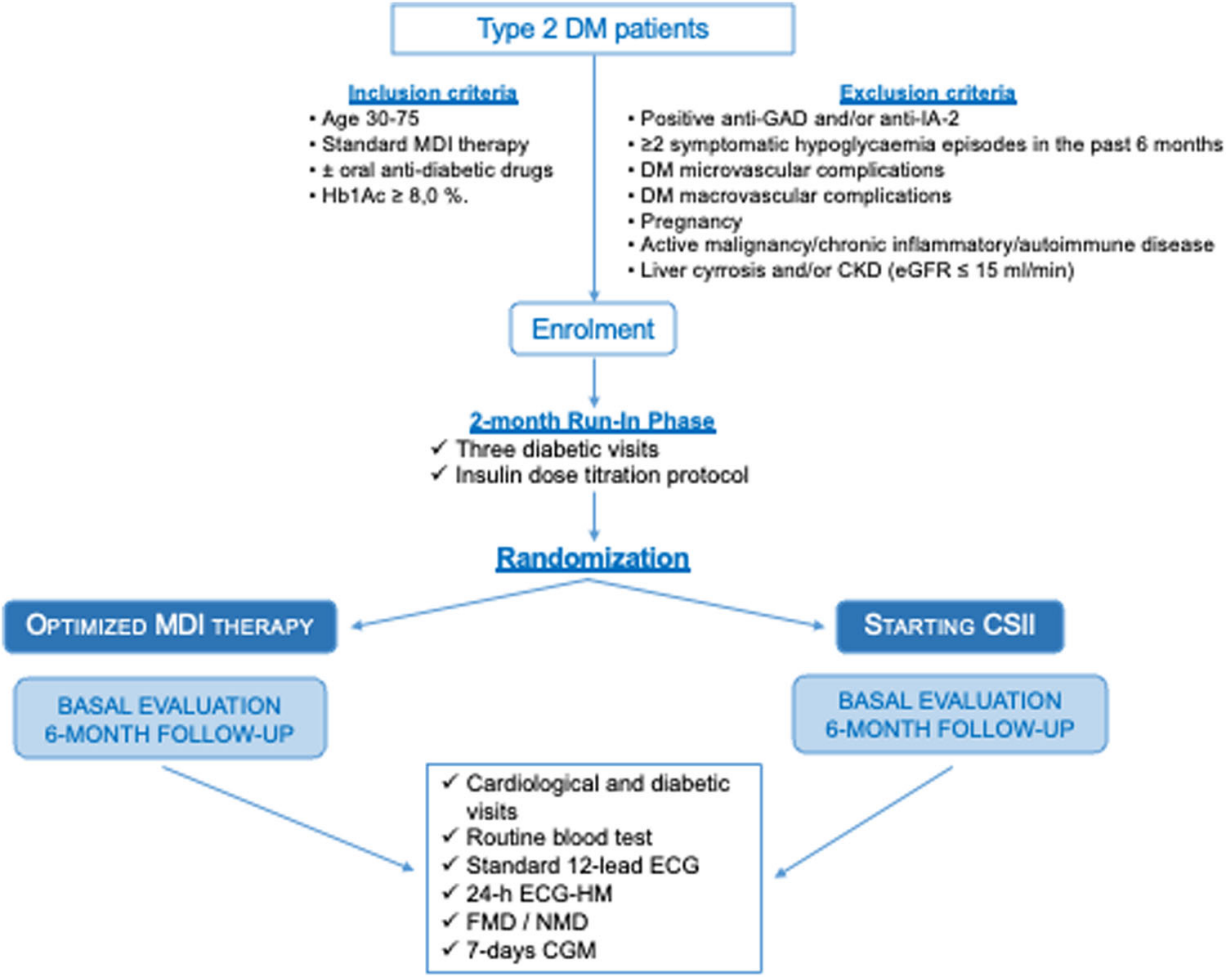
Flowchart of the trial protocol. CGM continuous glucose monitoring, CKD chronic kidney disease, CSII continuous subcutaneous insulin injection, DM diabetes mellitus, ECG electrocardiogram, eGFR estimated glomerular filtration rate, FMD flow-mediated dilation, GAD glutamic acid decarboxylase, HM Holter monitoring, IA-2 islet antigen-2, MDI multiple daily insulin injection, NMD nitrate-mediated dilation

The study protocol was drawn up in accordance with the requirements of Good Clinical Practice of the European Union and the current revision of the Declaration of Helsinki and was approved by the Ethics Committee of our Institution. Aims and methods of the study were clearly discussed with potentially eligible patients, who provided formal written informed consent to participate in the study. A flow chart of the study is shown in Fig. 1.

### Study design

Participants were enrolled at our Diabetes Care Unit. Before randomisation, patients underwent a 2-month run-in phase consisting of three clinical visits, designed to achieve optimal insulin injection treatment. During this period, insulin treatment was progressively increased according to a standardised titration protocol to achieve pre-prandial and post-prandial target ranges (fasting and pre-prandial 70–110 mg/dl and 2-h post prandial less than 180 mg/dl) of blood glucose levels, allowing adjustments of both basal and bolus insulin. The total dose increase was targeted at 10–40% above the basal dose of insulin. Patients were treated with both long-acting (Glargine or Detemir) and rapid-acting (Lispro, Aspart, or Glulisine) analogues of insulin. Patients were allowed to use insulin pens during the study for the administration of either rapid-acting or long-acting insulin analogues and injection preference was left to the Investigator’s standard clinical practice.

After the 2-month run-in period, patients were randomized to continue with multi-injection insulin treatment or pump treatment. Randomization was performed according to a computer-generated table of casual numbers, with even and odd figures assigned to CSII and MDI, respectively, without any stratification/block due to the low number of subjects expected. Since the study was open and based on results of objective glucose level data (obtained by CGM), diabetologists who enrolled and randomized patients could also be involved in the assessment of CGM data.

After randomization, patients assigned to pump treatment underwent a training period of up to 3 weeks after the end of the run-in phase to allow an optimal management of therapy. In both groups, treatment could be further adjusted according to glycemic data. Other types of anti-diabetic drugs were continued, but no adjustment of the dose was allowed.

Pump treatment was performed with the Medtronic 640 system (Medtronic Inc. Minneapolis, MN, USA).

### Study investigations

All patients underwent the following tests at baseline and after 6 months of treatment: 1) clinical cardiological and diabetes visit; 2) standard clinical chemistry tests and glycated haemoglobin (HbA1c) measurements; 3) resting 12-lead electrocardiogram (ECG); 4) seven-day continuous glucose monitor (CGM); 5) 24-h ECG Holter monitoring (HM); 6) flow-mediated dilation (FMD) and nitrate-mediated dilation (NMD) of the brachial artery.

### Continuous glucose monitoring

Continuous glucose monitoring (CGM) was performed for 7 days using the Medtronic iPro2 system (Medtronic, Inc. Minneapolis, MN, USA). CGM measures interstitial glucose at 5-min intervals, and patients were instructed to perform the required sensor calibration procedure four times a day according to the Manufacturer’s instructions. Data from the pump and blood glucose meter were uploaded with the Medtronic CareLink Therapy Management Software. Masked CGM data were obtained with Medtronic iPro2, with glucose data recorded over 7 days before randomisation and on completion of 6-month randomised treatment.

During the trial participants in the CSII arm were not allowed to use personal CGM but they were instructed to check blood glucose levels as those in the MDI arm.

The following parameters were obtained from the glucose recordings: 1) mean 24-h glucose concentration; 2) the standard deviation (SD) and coefficient of variation (CV) of interstitial blood glucose measurements; 3) time in range (TIR, i.e. the percentage of time with glucose levels between 70 and 180 mg/dL); 4) time above range 1 and 2 (TAR-1 and TAR-2), as a measure of hyperglycemic burden, defined as the percentage of time with glucose levels between 181–250 mg/dL and >250 mg/dL, respectively; 5) time below range 1 and 2 (TBR-1 and TBR-2), as a measure of hypoglycemic burden, defined as the percentage of time with glucose levels between 54–69 mg/dL and <54 mg/dL, respectively [20].

### Peripheral vasodilator function

Peripheral vasodilator function was assessed by the same expert operator using methods previously described in detail [13, 14, 21–24]. Patients were invited to refrain from exercise, smoking, alcoholic drinks and caffeine use in the 3 h preceding the test. Usual drugs were permitted as they remained constant throughout the study.

Endothelium-dependent vasodilatation was assessed by measuring flow-mediated dilatation (FMD). Briefly, subjects rested in the supine position for at least 10 min in a warm, quiet room (22 °C to 24 °C) before testing. A 10-MHz multi-frequency linear array probe and a high-resolution ultrasound machine were used to acquire images of the right brachial artery. Brachial artery diameter was measured throughout the whole test using a totally automated system that automatically identifies the internal edges of the vessel and tracks the walls of the artery via the brightness intensity of the walls vs. the lumen of the vessel (Cardiovascular Suite, Quipu srl, Pisa, Italy). The software provides a diameter measurement every second throughout the test. A mechanical support keeps the probe in a fixed position throughout the whole examination. After acquisition of baseline images of the brachial artery, a forearm cuff, positioned 1 cm under the antecubital fossa, was inflated to 250 mmHg and released after 5 min, thus resulting in forearm reactive hyperaemia. FMD was calculated as the maximum percent change of the brachial artery diameter during hyperaemia compared to baseline.

After recovery of brachial artery diameter to basal values, endothelium-independent vasodilatation was assessed by measuring nitrate-mediated dilatation (NMD). To this aim, 25 μg of sublingual glyceryl trinitrate was administrated and NMD was measured as the maximum percent change of the brachial artery diameter compared to the basal diameter.

### Electrocardiogram Holter monitoring

Twenty-four-hour ECG Holter recordings were performed using 3-channel recorders (Schiller Medilog AR4, Milan, Italy) and monitoring the ECG leads CM5-CM1 and modified aVF [25]. The recordings were analyzed by an expert operator using the Medilog Darwin‐2 system (Schiller Medilog).

Cardiac autonomic function was assessed by measuring HRV both in the time- and frequency-domain, using previously described methods [26, 27].

Time-domain HRV parameters included: SDNN (standard deviation of all RR intervals); SDNNi (mean of the standard deviations of RR intervals of all 5 min segments in the recording), RMSSD (the square root of the mean of the sum of the squares of differences between adjacent RR intervals), pNN50 (percent of consecutive pairs of adjacent RR intervals differing by more than 50 ms in the entire recording) and the triangular index (the ratio between the total number of RR intervals in the 24 h and the number of RR intervals with the modal value).

Frequency-domain HRV parameters, derived from power spectrum analysis of RR intervals by fast Fourier transform, included the amplitudes of RR oscillations in the range of very low frequency (VLF; 0.033–0.04 Hz), low frequency (LF; 0.04–0.15 Hz) and high frequency (HF; 0.15–0.40 Hz).

### Statistical analysis

The primary end-point of the study was endothelium-dependent vascular dilator function, i.e., FMD. Based on our previous experience, FMD in T2DM patients is around 4.5%, with a standard deviation of 1.2% [13]. Thus, we calculated that, to have an 80% power to detect as significant (at a two-tailed *p* value < 0.05) a between-group difference in FMD of 1.25%, we needed to enrol 15 patients per group.

Continuous variables were compared by independent t-test or Mann-Whitney test, as indicated, whereas Fisher exact test was applied to compare proportions. A repeated measure analysis of variance was applied to assess whether changes of variables at follow-up compared baseline differed between the 2 groups.

Correlations between percent changes in the main GV parameters (SD, CV and TIR) and percent changes in vascular function (FMD and NMD) at follow-up compared to baseline were assessed by Spearman correlation test.

The SPSS 28.0 statistical software (SPS, Florence, Italy) was used to perform statistical analyses. A *p* value < 0.05 was required for statistical significance.

## Results

Overall, 21 patients were enrolled in the study: 12 patients were randomized to insulin pump treatment (Group 1) and 9 patients were assigned to continue multi-injection insulin treatment (Group 2). The main demographic and clinical characteristics of the whole population and the 2 groups of patients are summarized in Table 1. As shown, patients randomized to Group 1 were younger compared to those of Group 2 (53.9 ± 9.6 vs. 65.4 ± 7.6 years, respectively, *p* = 0.008); but there were no differences in gender, cardiovascular risk factors and non-insulin anti-diabetic therapy. Body weight did not differ significantly between the 2 groups at baseline (83.9 ± 16 vs. 85.9 ± 17 Kg; *p* = 0.79) and showed no significant changes in both groups at follow-up (84.2 ± 14 vs. 84.0 ± 16 Kg; *p* = 0.98; *p* for changes = 0.23).

**Table 1:** Main clinical characteristics of patients enroled

	Group 1 (*n* = 12)	Group 2 (*n* = 9)	*p* value
Age (years)	53.9 ± 9.6	65.4 ± 7.6	0.008
Male sex	8 (66.7%)	6 (66.7%)	0.68
Cardiovascular risk factors			
Hypertension	6 (50.0%)	8 (88.9%)	0.08
Dyslipidaemia	9 (75.0%)	8 (88.9%)	0.41
Active smoking	3 (25.0%)	2 (22.2%)	0.65
Body weight (Kg)	83.9 ± 16	85.9 ± 17	0.79
BMI (Kg/m^2^)	28.7 ± 5.6	29.9 ± 5.3	0.62
SCORE-2 diabetes (%)	9.1 ± 5	12.0 ± 5	0.12
Non-insulin anti-diabetic drugs			
Metformin	5 (41.7%)	4 (44.4%)	1.00
SGLT2 inhibitor	3 (25.0%)	4 (44.4%)	0.40

*BMI* body mass index, *CV* cardiovascular, *SGLT2* sodium-glucose co-transporter 2

Baseline and follow-up data of diabetes status are summarized in Table 2. The daily dose of insulin and glycated hemoglobin serum levels (Hb1AC) did not differ significantly between the 2 groups, both at baseline (*p* = 0.17 and *p* = 0.08, respectively) and at follow-up (*p* = 0.22 and *p* = 0.36, respectively).

**Table 2:** Glycated hemoglobin, insulin dose and main parameters of continuous glucose monitoring in the 2 groups of patients

		Group 1 (*n* = 9)	Group 2 (*n* = 8)	*p* value	*p* for changes
Hb1AC [% (mmol/mol)]	baseline	8.0 ± 1.0 (64 ± 8)	8.9 ± 1.2 (74 ± 13)	0.08	0.50
	follow-up	7.9 ± 1.0 (63 ± 8)	8.9 ± 1.6 (63 ± 17)	0.36	
Total daily insulin dose (IU/Kg)	baseline	0.56 ± 0.20	0.71 ± 0.26	0.17	0.43
	follow-up	0.63 ± 0.15	0.74 ± 0.24	0.22	
Mean interstitial blood glucose level (mg/dL)	baseline	159.7 ± 18	165.0 ± 16	0.52	0.25
	follow-up	146.8 ± 8	161.3 ± 18	0.051	
SD of interstitial blood glucose level (mg/dL)	baseline	57.4 ± 11.1	62.3 ± 10.1	0.37	0.20
	follow-up	43.2 ± 10.4	56.8 ± 16.0	0.052	
CV of interstitial blood glucose level (%)	baseline	35.7 ± 3.3	37.6 ± 3.4	0.27	0.25
	follow-up	29.2 ± 5.7	35.1 ± 8.4	0.11	
Time in range (%)	baseline	69.0 ± 15.3	61.3 ± 11.0	0.26	0.84
	follow-up	71.0 ± 10.1	64.0 ± 12.2	0.22	
Time above range-1 (%)	baseline	25.1 ± 13.1	28.5 ± 11.0	0.57	0.41
	follow-up	224.4 ± 6.9	28.5 ± 10.6	0.18	
Time above range-2 (%)	baseline	4.4 ± 3.7	7.3 ± 2.9	0.11	0.76
	follow-up	5.2 ± 4.2	5.8 ± 4.5	0.81	
Time below range-1 (%)	baseline	1.2 ± 1.6	2.3 ± 1.5	0.18	0.32
	follow-up	1.2 ± 1.0	1.6 ± 0.9	0.40	
Time below range-2 (%)	baseline	0.2 ± 0.7	0.8 ± 0.9	0.18	0.94
	follow-up	0.1 ± 0.3	0.1 ± 0.4	0.93	

*Hb1Ac* glycated haemoglobin, *SD* standard deviation, *CV* coefficient of variation

Complete CGM could be obtained both at baseline and at 6-month follow-up in 17 patients (81%), 9 of Group 1 and 8 of Group 2. As shown in Table 2, CGM parameters at baseline did not differ significantly between the 2 groups. At follow-up no changes were observed for several CGM variables, including TIR, TAR-1, TAR-2 and TBR-1. However, a significant reduction was observed in the whole population of patients in mean blood glucose levels (*p* = 0.047), both SD (*p* = 0.01) and CV (*p* = 0.016) of glucose blood levels, and TBR-2 (*p* = 0.039), but there were no differences in the changes of these parameters between the 2 groups (Table 2). No episodes of symptomatic or severe hypo-glycemia or diabetic ketoacidosis were recorded during the study.

The main results of peripheral vascular dilator function are shown in Table 3. Neither flow-mediated dilatation (FMD) nor nitrate-mediated dilatation (NMD) showed significant changes at follow-up in the whole population, and no differences were observed between patients of Group 1 and Group 2 in both basal and follow-up values, even after correction for age. Correlation analyses showed no significant relation between percent changes in the main GV parameters (SD, CV, TIR) and percent changes in FMD and NMD at follow-up compared to basal values (Table 4).

**Table 3:** Flow mediated dilatation (FMD) and nitrate mediated dilatation (NMD) at baseline ad at follow-up

		Group 1 (*n* = 12)	Group 2 (*n* = 9)	*p* value	*p* for changes
Flow mediated dilatation (%)	baseline	6.0 ± 3.8	6.1 ± 7.0	0.96	0.35*
	follow-up	6.7 ± 2.5	4.6 ± 2.0	0.51	
Nitrate mediated dilatation (%)	baseline	8.6 ± 4.7	7.1 ± 3.4	0.43	0.81†
	follow-up	8.9 ± 3.1	7.0 ± 3.0	0.18	

*p = 0.42 after correction for age, †*p* = 0.40 after correction for age

**Table 4:** Correlations between changes in glucose variability parameters and vascular function

	% FMD changes		% NMD changes	
	rho	*p*	rho	*p*
% SD changes	0.21	0.41	0.24	0.34
% CV changes	0.06	0.81	0.18	0.50
% TIR changes	−0.15	0.55	0.15	0.57

*CV* coefficient of variation, *FMD* flow-mediated dilation, *NMD* nitrate-mediated dilation, *SD* standard deviation, *TIR* time in range

HRV parameters, both in the time- and the frequency-domain, also showed no significant differences between the 2 groups, both at baseline and at follow-up, with no changes compared to baseline (Table 5).

**Table 5:** HRV parameters^a^ at baseline and at 6-month follow-up

		Group 1 (*n* = 12)	Group 2 (*n* = 9)		*p* value	*p* for changes
Mean HR (bpm)	baseline	75 ± 11		74 ± 14	0.86	0.76
	follow-up	76 ± 9		75 ± 11	0.70	
SDNN (ms)	baseline	106.8 ± 31.0		100.2 ± 34.2	0.65	0.52
	follow-up	107.3 ± 39.1		93.4 ± 32.4	0.40	
SDNNi (ms)	baseline	37.5 ± 11.9		42.7 ± 20.9	0.48	0.17
	follow-up	37.3 ± 10.0		38.1 ± 18.5	0.89	
RMSSD (ms)	baseline	21.3 ± 10.2		31.8 ± 24.5	0.20	0.43
	follow-up	21.3 ± 10.3		28.2 ± 18.4	0.29	
pNN50 (%)	baseline	4.5 ± 6.7		8.8 ± 10.0	0.26	0.61
	follow-up	4.8 ± 5.8		8.1 ± 9.9	0.36	
VFC index	baseline	28.9 ± 10.3		26.4 ± 10.2	0.59	0.41
	follow-up	29.9 ± 10.5		24.4 ± 13.7	0.21	
VLF (ms)	baseline	28.5 ± 8.4		29.2 ± 13.0	0.89	0.28
	follow-up	27.5 ± 7.1		26.2 ± 13.7	0.77	
LF (ms)	baseline	17.3 ± 6.8		19.1 ± 12.7	0.70	0.17
	follow-up	16.2 ± 6.1		16.5 ± 10.6	0.93	
HF (ms)	baseline	10.5 ± 5.6		12.9 ± 8.7	0.48	0.26
	follow-up	10.4 ± 5.1		10.3 ± 6.7	0.96	

^a^See Methods for definition of HRV parameters; *HR* Heart Rate

## Discussion

Our data show that in T2DM patients, insulin pump therapy can be performed safely, achieving a similar medium-term glycemic control compared to MDI and without any significant adverse effect on the cardiovascular system. Of note, since the use of insulin pump is low in Type 2 diabetes in Italy, our results provide helpful information to support its use in this population of patients.

Uncontrolled T2DM has long been known to be associated with increased cardiovascular events [4], and several studies have shown that increased variability of glucose blood levels is also associated with increased cardiovascular risk, even in patients with sufficiently controlled blood glucose and HbA1c levels [28]. In particular, increased episodes of hypoglycaemia have been suggested to portend an ominous cardiovascular prognosis, likely resulting in strong adrenergic activation and, therefore, cardiac sympatho-vagal imbalance [8–10]. Furthermore, a suboptimal control of glucose values may also result in an impairment of vascular function, in particular endothelium-mediated dilation function [13–15], which may also contribute to the increased risk of cardiovascular events [29].

Treatment of T2DM in insulin-dependent patients has long been based on multiple subcutaneous injections of insulin associated with multiple self-assessments of blood glucose levels. However, in the last few decades, CSII pumps have been developed, with the advantage of removing the need for multiple insulin injections throughout the day. Furthermore, novel devices, such as glucose sensors, have recently been developed to continuously measure glucose levels throughout the day, which alleviates the need to measure capillary blood glucose multiple times throughout the day [30]. Importantly, several studies have shown comparable control of glycaemic status in patients treated with CSII compared to standard MDI [31–34].

In this study, we aimed to investigate whether these two methods of treatment might result in different effects on the cardiovascular system, specifically on peripheral vascular dilation (both endothelium-independent and endothelium-dependent) function and cardiac autonomic function. Furthermore, we also aimed to assess whether possible differences might have been related to different control of glycaemic levels and status.

Our data show that the 2 forms of treatment seem to be comparable in the control of glycaemic status as well as on the assessed cardiovascular outcome. Of note, despite no changes were observed in HbA1C, GV showed a significant improvement at follow-up compared to baseline in the whole population of patients, as indicated by the reduction of SD and CV of interstitial glucose levels, as well as TBR-2. However, there were no differences in the changes between the 2 groups. Nevertheless, this better GV profile did not result in any significant change at follow-up of both peripheral dilation function (as assessed by both FMD and NMD) and cardiac autonomic function (as assessed by 24-h HRV), which also showed no significant differences between the 2 groups both at baseline and at follow-up.

### Limitations of the study

Some limitations of our study need to be acknowledged. Firstly, we were unable to enrol the planned number of patients due to COVID-19 pandemic lockdown; thus, we could eventually include in the study only a small population of patients with some imbalance of the 2 groups; accordingly, our data need further assessment in larger populations. Secondly, due to the small sample population, randomization was suboptimal, with Group 1 patients being younger than Group 2 patients; however, there were no other significant differences in both clinical and metabolic data between the 2 groups. Finally, our study was limited to 6 months of follow-up and therefore we cannot make conclusions about whether differences between the 2 groups, both in metabolic glycemic control and cardiovascular effects, might emerge during longer periods of follow-up.

In conclusion, in this randomized small study we show that, in T2DM patients, insulin pump therapy achieves a similar medium-term glycemic control compared to MDI, without any significant adverse effect on the cardiovascular system.

### Supplementary information

Consort 2010 checklist

## Data Availability

The data that support the findings of this study are available from the corresponding author upon reasonable request.
